# High-Dose Immunoglobulin Therapy for Multiple Thromboembolism in Persisting Heparin-Induced Thrombocytopenia

**DOI:** 10.7759/cureus.55747

**Published:** 2024-03-07

**Authors:** Yasushi Kudo, Koki Suzuki, Shota Maezawa, Ryota Seo, Takashi Irinoda

**Affiliations:** 1 Department of Emergency Medicine, Osaki Citizen Hospital, Miyagi, JPN

**Keywords:** internal iliac artery thrombus, pulmonary artery thrombus, direct oral anticoagulant therapy, aortic mural thrombus, intravenous immunoglobulin (ivig), auto-immune heparin induced thrombocytopenia, heparin induced thrombocytopenia(hit)

## Abstract

This report presents a case of an 81-year-old male with acute respiratory distress syndrome secondary to aspiration pneumonia who developed heparin-induced thrombocytopenia (HIT). His platelet count remained persistently low despite discontinuing unfractionated heparin and initiating intravenous argatroban. Multiple thromboembolisms, including a new aortic mural thrombus in the descending aorta, were observed on contrast-enhanced computed tomography (CT), resulting in a diagnosis of autoimmune HIT (aHIT). Subsequent high-dose intravenous immunoglobulin (IVIG) therapy substantially improved the platelet count and resolved thromboembolisms. This case is notable owing to the improvement of aHIT complicated by multiple thromboembolisms, including an aortic mural thrombus, following high-dose IVIG therapy. In recent years, a growing number of reports have documented the effectiveness of high-dose IVIG therapy for aHIT. However, reports on whether high-dose IVIG therapy could improve an aortic mural thrombus complicating aHIT are lacking. The successful use of high-dose IVIG therapy in the current case highlights its potential efficacy in treating aHIT complicated by multiple thromboembolisms. Further studies are required to clarify the role of IVIG in the management of aHIT with thromboembolism.

## Introduction

Heparin-induced thrombocytopenia (HIT) is a disorder characterized by consumptive thrombocytopenia and systemic arterial and venous thrombosis triggered by exposure to heparin. The standard treatment for HIT involves heparin discontinuation and initiation of alternative anticoagulation therapy [1]. However, in cases where no improvement is achieved, treatments such as high-dose intravenous immunoglobulin (IVIG) therapy and plasma exchange are considered, although their application is yet to be standardized. A subtype of HIT, known as autoimmune HIT (aHIT), is particularly refractory to the standard treatment. Recent years have seen an increase in reports highlighting the effectiveness of high-dose immunoglobulin therapy in managing aHIT [2]. However, to the best of our knowledge, there are no confirmed reports of the effectiveness of high-dose IVIG therapy in aHIT cases complicated by an aortic mural thrombus. In the current report, we present a case of aHIT complicated by multiple thromboembolic events, including an aortic mural thrombus, which was successfully treated with high-dose IVIG therapy.

## Case presentation

**Figure 1:**
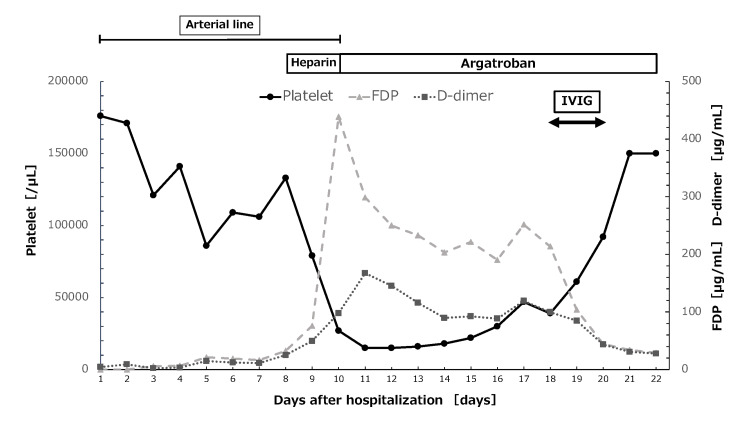
Trends in platelet, FDP, and D-dimer levels after hospitalization and the course of drugs used. FDP, fibrin degradation products; IVIG, intravenous immunoglobulin Reference value: Platelet 158,000-348,000/μL, FDP 0-5 μg/mL, D-dimer 0-1 μg/mL

An 81-year-old male presented to the emergency department with respiratory failure and was diagnosed with acute respiratory distress syndrome secondary to aspiration pneumonia. He underwent oral intubation, along with the insertion of an arterial line for continuous blood pressure monitoring. A normal saline solution with unfractionated heparin (1 U/mL) was used as an arterial line flush solution, continuously infused at 3 mL/h under a pressure of 300 mmHg. After admission to the intensive care unit, his respiratory status improved, leading to extubation on day five. On day eight, he developed atrial fibrillation, and continuous intravenous unfractionated heparin (10,000 U/day) was initiated. Two days after initiating heparin therapy, his platelet count decreased from 79,000/μL to 27,000/μL. Fibrin degradation products (FDP) and D-dimer were markedly elevated (Figure 1).

**Figure 2:**
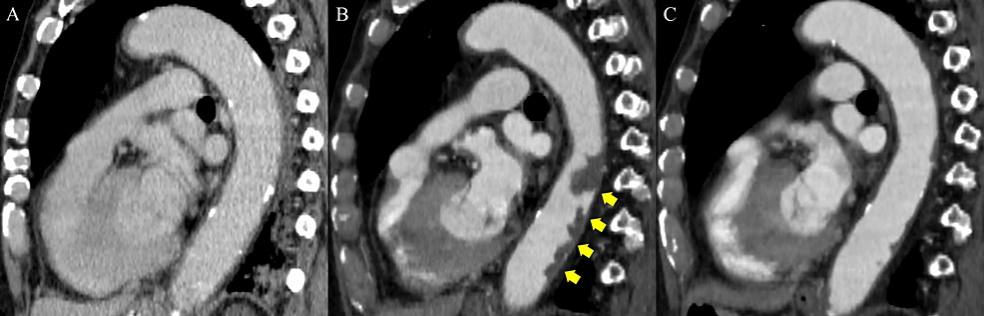
The descending aorta in contrast-enhanced CT A: CT scan upon admission, showing the descending aorta. B: CT after diagnosis of persisting HIT, revealing a mural thrombus in the descending aorta (yellow arrows). C: CT following high-dose IVIG therapy, showing near-complete resolution of the aortic mural thrombus. HIT, heparin-induced thrombocytopenia, IVIG, Intravenous immunoglobulin

Thrombotic occlusion of the arterial line was observed the same day. The patient had no prior history of heparin exposure except for the flushing solution. The 4Ts score was 7 points (2 points for thrombocytopenia, 2 points for timing of platelet count fall, 2 points for thrombosis, and 1 point for possible other causes for thrombocytopenia), thereby suggesting a high probability of HIT. Based on the clinical course and the 4Ts score, HIT was suspected, resulting in the discontinuation of continuous intravenous heparin and the initiation of continuous intravenous argatroban. Argatroban was started at 0.7 μg/kg/min and adjusted to maintain the activated partial thromboplastin time within 1.5-3 times the baseline value. Based on the latex agglutination test (HemosIL® HIT-Ab (PF4-H), normal range <1.0 U/mL, upper limit 4.9 U/mL), the level of anti-platelet factor 4 (PF4)-heparin complex antibodies was > 5 U/mL, confirming HIT antibody positivity. Despite treatment, the patient’s platelet count remained persistently low. On day 18, contrast-enhanced computed tomography (CT) was performed, revealing a new mural thrombus in the descending aorta (Figure 2A, 2B), right pulmonary artery thrombus, and left internal iliac artery thrombus.

A diagnosis of persisting HIT was established, and the patient received high-dose IVIG therapy (1 g/kg/day) for two days. Following IVIG administration, his platelet count increased from 39,000/μL to 150,000/μL. On day 29, continuous intravenous argatroban was discontinued, and oral edoxaban (30 mg) was initiated. A follow-up contrast-enhanced CT on day 57 revealed near-complete resolution of the aortic mural thrombus (Figure 2C), right pulmonary artery thrombus, and left internal iliac artery thrombus. The patient did not develop arterial embolism owing to the mural thrombus and was transferred to a rehabilitation facility without complications on day 68.

## Discussion

HIT is a disorder caused by exposure to heparin, resulting in thrombocytopenia and systemic arterial and venous thromboembolism. Upon administration, heparin forms a complex with PF4, with anti-PF4-heparin complex antibodies (HIT antibodies) subsequently generated against this complex. The formation of an immune complex between these HIT antibodies and the PF4-heparin complex, which binds to FcγRⅡa on the platelet membrane, activates platelets and triggers the coagulation cascade [3,4]. This activation accelerates thrombin generation, leading to consumptive thrombocytopenia and systemic arterial and venous thromboembolism. Therefore, in the treatment of HIT, in addition to discontinuing heparin, it is necessary to administer therapeutic doses of alternative anticoagulants [5].

Two methods are available for measuring HIT antibodies: immunological and functional assays. The latex agglutination test performed in the current case falls under the category of immunological assays. Immunological assays for HIT antibodies have high sensitivity but poor specificity [6]. Therefore, for a definitive diagnosis, functional assays for HIT antibodies are preferable. However, functional assays for HIT antibodies are not commercially available in Japan, making them difficult to implement. Although the possibility of a false positive for HIT antibodies cannot be completely ruled out, considering the clinical course, the diagnosis of HIT in the present case can be deemed accurate.

There is a subtype of HIT known as aHIT, which includes delayed-onset HIT, persisting HIT, spontaneous HIT syndrome, flush heparin HIT, fondaparinux-associated HIT, and severe HIT with overt disseminated intravascular coagulation. In the current case, a diagnosis of persisting HIT was established, which is defined as “HIT that persists for more than one week despite the discontinuation of heparin.” In aHIT, platelet activation by HIT antibodies can occur independently of heparin, frequently rendering the standard HIT treatment of heparin discontinuation and initiation of alternative anticoagulation therapy insufficient [7].

In 1989, Frame et al. first documented the efficacy of immunoglobulin therapy in the treatment of HIT [8]. Since then, the opinions on immunoglobulin therapy for HIT remain ambivalent, hindering its establishment as a standard treatment. With the pathophysiology of aHIT becoming more evident, a growing number of reports have documented the effectiveness of high-dose immunoglobulin therapy in aHIT in recent years [9]. Considering the mechanism of action of high-dose immunoglobulin therapy, it is speculated that the administered IgG competes with HIT antibodies, thereby inhibiting platelet activation mediated by HIT antibodies through FcγRⅡa on the platelet membrane [10]. Considering the pathophysiology of aHIT and the mechanism of action of high-dose immunoglobulin therapy, immunoglobulin therapy for aHIT seems pretty rational.

In the current case report, the patient had no previous exposure to heparin and was sensitized by a heparin-containing flush solution used in the arterial line. Moreover, despite discontinuing heparin and initiating argatroban, the patient’s thrombocytopenia and high levels of FDP and D-dimer persisted. Additionally, contrast-enhanced CT revealed new thrombi, including an aortic mural thrombus, a right pulmonary artery thrombus, and a left internal iliac artery thrombus. These findings led to a diagnosis of persisting HIT and treatment with high-dose immunoglobulin therapy. The high-dose immunoglobulin therapy markedly improved thrombocytopenia and thromboembolism.

Herein, the patient also presented with aortic mural thrombus as part of the multiple thromboembolism. Aortic mural thrombus, a rare condition, can be managed using various treatment approaches, including anticoagulation therapy, thrombolytic therapy, and thrombectomy. However, a consensus on a standard treatment approach is yet to be established [11-13]. Currently, there are no confirmed reports of an aortic mural thrombus complicating aHIT that improved with high-dose immunoglobulin therapy. In the current case, the improvement in multiple thromboembolism, including aortic mural thrombus, could be attributed to the control of aHIT by high-dose immunoglobulin therapy, anticoagulation therapy, and the body’s fibrinolytic mechanisms.

## Conclusions

High-dose immunoglobulin therapy may be effective in treating aHIT complicated by multiple thromboembolisms, including aortic mural thrombus. In aHIT cases, the standard HIT treatment, such as heparin discontinuation and initiation of alternative anticoagulation therapy, often fails to address thrombocytopenia and systemic thromboembolism effectively. This underscores the need for alternative approaches. The presence of aortic mural thrombus, which can lead to various fatal ischemic complications, further emphasizes the urgency for effective treatment strategies. Therefore, promptly recognizing aHIT and initiating high-dose IVIG therapy could be critical. Further research is essential to comprehensively clarify the effectiveness of high-dose immunoglobulin therapy in aHIT cases accompanied by thromboembolism.
